# Ocular Symptoms as a Marker of Dysautonomia in Long-COVID Patients: A Cross-Sectional Analysis

**DOI:** 10.3390/brainsci16020135

**Published:** 2026-01-27

**Authors:** Sakina Qazi, Chloe Shields, Kimberly Cabrera, Jane Nguyen, Pragnya Rao Donthineni, Normila Barthelemy, Araliya Gunawardene, Paula Sepulveda-Beltran, Leonardo Tamariz, Anat Galor

**Affiliations:** 1 Surgical and Research Services, Miami Veterans Administration Medical Center, Miami, FL 33125, USA; sqazi@med.miami.edu (S.Q.); cxs1034@med.miami.edu (C.S.); kcabrera@s.icom.edu (K.C.); drpragnyarao@lvpei.org (P.R.D.); nxb527@med.miami.edu (N.B.); gunawardenearaliya@gmail.com (A.G.); paulasb94@gmail.com (P.S.-B.); ltamariz@med.miami.edu (L.T.); 2 Bascom Palmer Eye Institute, University of Miami, Miami, FL 33136, USA; jtn40@med.miami.edu

**Keywords:** ocular pain, dry-eye disease, dysautonomia, long-COVID

## Abstract

**Background/Objectives**: Post-coronavirus syndrome (long-COVID) refers to a multi-systemic range of symptoms that follows acute SARS-CoV-3 infection. Long-COVID has been linked with autonomic neuropathy as well as dry-eye disease (DED), an umbrella term that includes a variety of ocular symptoms and signs. Despite these associations, little is known about the co-occurrence of DED and dysautonomia symptoms in individuals with long-COVID. This study aims to examine relationships between dysautonomia and ocular symptoms in a long-COVID patient population. **Methods**: Cross-sectional study of 162 veterans with long-COVID. The Composite Autonomic Symptom Score-31 (COMPASS-31) assessed dysautonomia symptoms, and the NASA lean test and heart-rate variability metrics captured dysautonomia signs. Dry-eye disease (DED) symptoms were measured with the 5-Item Dry-Eye Questionnaire (DEQ5) and the Ocular Surface Disease Index (OSDI), while ocular pain intensity and neuropathic pain descriptors were evaluated using a Numerical Rating Scale (NRS) and select questions from the Neuropathic Pain Symptom Inventory modified for the Eye (NPSI-Eye), respectively. **Results**: Most participants (78%) reported DED symptoms (DEQ5 ≥ 6). Nearly all COMPASS-31 domains were associated with DED symptoms, with the strongest correlation observed between the OSDI and pupillomotor scores (r = 0.67, *p* < 0.001). Among the autonomic signs, the strongest associations were observed between the change in systolic and diastolic blood pressure from baseline to 8 min and ocular pain triggered by temperature (r = −0.44 and r = −0.48, respectively, *p* < 0.01 for both). On linear regression analyses, pupillomotor and secretomotor symptoms remained positively associated with DED symptoms, while autonomic signs were most closely related to ocular pain metrics, with fluctuating blood pressure changes during orthostasis relating to neuropathic symptoms. **Conclusions**: DED symptoms, including ocular pain intensity, relate to autonomic symptoms in a long-COVID cohort. While associations with autonomic signs were less consistent, these data suggest that subtle autonomic variability relates to ocular pain in the long-COVID setting.

## 1. Introduction

Post-coronavirus syndrome (long-COVID) refers to a range of sequelae that follow acute severe acute respiratory syndrome coronavirus-2 (SARS-CoV-2) infection. Multiple clinical definitions have been developed, but the World Health Organization (WHO) defines a “post-COVID-19 condition” as “symptoms occurring at least three months after probable or confirmed SARS-CoV-2 infection [1,2].” Studies have reported that 12–14% of patients with COVID-19 develop persistent symptoms which include cardiopulmonary symptoms such as chest pain and difficulty breathing [3], musculoskeletal symptoms such as myalgias, sensory symptoms such as ageusia or anosmia, neurological symptoms such as brain fog [4], autonomic symptoms such as tachycardia [5] and general symptoms such as fatigue [6,7]. The frequency of long-COVID may be even higher in certain populations, with one study reporting an incidence of nearly 60% in a national Veterans Affairs (VA) healthcare system population [8].

Of all symptoms, dysautonomia symptoms are among the most disabling and widely described in long-COVID [9]. The autonomic nervous system (ANS) is critical in maintaining homeostasis, and the distribution of autonomic nerves throughout the body creates multiple sites for potential dysfunction. Long-COVID dysautonomia can thus present with syndromic sets of symptoms and signs, depending on which components of the ANS are inappropriately activated or inhibited [10]. For example, many patients present with symptoms of orthostatic intolerance (OI), such as light-headedness and palpitations, with orthostatic hypotension and/or postural orthostatic tachycardia syndrome (POTS) confirmed on testing in 28% to 74% of long-COVID populations, as reported across multiple studies [11,12,13,14,15]. Other autonomic symptoms include gastrointestinal (e.g., bloating, diarrhea or constipation), sudomotor (e.g., increased/decreased sweating), and visual (e.g., impaired accommodation) complaints [16,17], again with objective signs (e.g., increased latency and decreased duration of pupil contraction, decreased sweat output on Quantitative Sudomotor Axon Reflex Test) supporting these complaints [18,19].

Along with ANS dysfunction, COVID infection has also been linked to aspects of dry-eye disease (DED). One study examined 228 individuals, 4–12 weeks after their hospitalization for COVID, and 109 controls who had no history of COVID. A total of 21.5% of cases reported at least one new-onset ocular symptom, most commonly blurry vision, from 14% who reported ocular symptoms prior to their COVID diagnosis. Corneal staining (range 0–3) was more severe in the post-COVID group compared to the control group (0.60 ± 0.69 vs. 0.49 ± 0.68, *p* = 0.04), as were Meibomian gland abnormalities (plugging and meibum quality composite score, range 1–4, 1.14 ± 0.67 vs. 0.92 ± 0.68, *p* = 0.002) [20]. Longitudinal studies support the cross-sectional associations. One study followed a cohort of patients diagnosed with DED (n = 22) who developed COVID-19 and compared pre-COVID and post-COVID (4.61 ± 2.39 months after infection) eye exams. Using the LipiView, lipid layer thickness decreased (63.00 ± 22.40 → 52.86 ± 18.00 nm, *p* < 0.001) while Meibomian gland atrophy increased in both the upper (1.55 ± 0.73 → 1.75 ± 0.84, *p* = 0.01) and lower (1.30 ± 0.51 → 1.43 ± 0.73, *p* = 0.001) eyelids (range 0–4) [21]. These studies link COVID infection to DED symptoms and signs, at least transiently.

Despite associations of COVID-19 infection with dysautonomia and DED as separate entities, there is a paucity of research on the co-occurrence of DED and dysautonomia symptoms in individuals with long-COVID. Additionally, it is unclear which autonomic domains are most related to DED symptoms in the context of long-COVID. This study aims to bridge these knowledge gaps. Advancing knowledge of the eye–autonomic nervous system connection holds promise for developing more precise diagnostics and tailored therapies in long-COVID, with the goal of alleviating the symptoms and signs of this complex and potentially debilitating condition.

## 2. Materials and Methods

### 2.1. Study Population

This was a cross-sectional study that included a sample of veterans with long-COVID who were evaluated in the Long-COVID Clinic at the Miami Veterans Affairs (VA) Hospital [22]. Using convenience sampling, all patients seen in the clinic from November 2021 to June 2023 who met the WHO definition of long-COVID [1] and completed questionnaires regarding autonomic and ocular symptoms were included (n = 162). Of these, 74 also underwent in-office autonomic testing (NASA lean test and/or HRV testing). No individuals meeting these criteria were excluded from analysis. Symptom questionnaires and autonomic testing were not performed contemporaneously during clinical evaluation. The retrospective review of records was approved by the Miami VA Institutional Review Board (IRB number 3011.01).

### 2.2. Data Collection

Data on demographics, comorbidities, and medications were collected by chart review.

### 2.3. Assessment of Autonomic Function

#### 2.3.1. Dysautonomia Symptoms

All individuals graded their dysautonomia symptoms using the modified Composite Autonomic Symptom Score-31. This questionnaire addresses 6 autonomic domains with 31 questions: orthostatic intolerance, 4 items; vasomotor, 3 items; secretomotor, 4 items; gastrointestinal, 12 items; bladder, 3 items; and pupillomotor, 5 items. Each symptom is scored binomially as 0 if not present and 1 if present, with further quantification of symptom frequency (0 for rarely or never, 1 for occasionally or sometimes, 2 for frequently or a lot of the time, and 3 for constantly). Finally, symptom course is scored as 0 for improved or not present, 1 for stayed the same, 2 for gotten somewhat worse, and 3 for gotten much worse. The final score sums the raw domain scores multiplied by a weight factor, with a range of 0–100. This instrument has been validated as a measure of dysautonomia symptom severity [23,24].

#### 2.3.2. Dysautonomia Signs

Thirty-eight individuals underwent heart-rate variability (HRV) testing at rest, measured using an emWave Pro Plus device (Heartmath Inc., Boulder Creek, CA, USA) with a probe placed over the ear lobe for 2 min, with the patient instructed to stay still. The validity of the short-time-period measures using this device has been established [25]. Reported metrics include the root mean square of successive differences between normal heartbeats (RMSSD), a measure of beat-to-beat variance in heart rate (HR), and the standard deviation of interbeat intervals of normal sinus beats (SDNN), a measure of sympathetic and parasympathetic activity. HR oscillations were divided into low-frequency (Lf) and high-frequency (Hf) bands; the ratio of Lf to Hf power (Lf/Hf ratio) was reported as a measure of the ratio of sympathetic to parasympathetic activity.

Sixty-three individuals underwent the NASA lean test to evaluate the effect of standing on blood pressure (BP) and heart rate (HR). This test is validated and accurate when compared to the tilt table test [13]. BP and HR were measured with the participant supine for two minutes, then again at 1-min intervals for 10 min while standing leaning against a wall. Orthostatic hypotension (OH) was defined as a decrease of ≥20 mmHg in the systolic BP (SBP) and/or ≥10 mmHg in the diastolic BP (DBP) from the baseline supine value within 3 min of standing. Postural orthostatic tachycardia syndrome (POTS) was defined as an increase in HR of >30 bpm within 10 min of position change in the absence of orthostatic hypotension [17]. In addition, differences in SBP, DBP, pulse pressure (PP), and HR from baseline to 2-, 3-, 5-, 6-, 7-, 8-, 9-, and 10 min were recorded.

### 2.4. Assessment of Ocular Symptoms

All individuals graded their ocular symptoms on standardized questionnaires. Composite DED questionnaires included the 5 Item Dry-Eye Questionnaire (DEQ5) [26], range 0–22, and Ocular Surface Disease Index (OSDI), range 0–100 [27]. Ocular pain intensity was graded using a Numerical Rating Scale (NRS, range 0–10), a validated measure of pain intensity [28,29], with ratings for average pain over one week recall, worse pain over one week recall, and pain right now. Neuropathic ocular pain features were assessed using 4 questions from the Neuropathic Pain Symptom Inventory validated for the Eye (NPSI-Eye) [30]. These included questions regarding intensity of burning, and intensity of evoked pain to wind, light, and heat/cold, each rated on a scale of 0–10. Patients were divided into DED and non-DED symptom groups based on established DEQ5 cut-off values (no symptoms < 6, mild to severe symptoms 6–22) [26].

### 2.5. Data Analysis

Data were analyzed using SPSS 29.0 (SPSS Inc., Chicago, IL, USA). Descriptive statistics were used to summarize demographic and clinical data. Autonomic profiles were compared between individuals with and without DED symptoms using the independent sample *t*-test and Chi-square test, as appropriate. Baseline demographic and clinical characteristics were compared between individuals who underwent autonomic testing and those who did not to assess for potential selection bias. Repeated-measures ANOVA was performed to compare trends in BP and HR over time during the NASA lean test across DED symptom groups. Pearson correlations were used to examine relationships between ocular symptoms and autonomic parameters. Forward stepwise multiple regression analyses were performed to further understand these relationships, adjusting for potential confounders (i.e., demographics, medications, medical and ocular comorbidities). Sensitivity analyses were performed to assess the extent to which observed associations were driven by item overlap between the COMPASS-31 and ocular questionnaires, by repeating Pearson correlations and multivariable regression models using modified COMPASS-31 subscores excluding ocular-related items (item 9 of the secretomotor domain and the entire pupillomotor domain (items 27–31)). Missing data were addressed by reporting sample sizes for each analysis. In this paper, information was given on all variables being compared as opposed to correcting the *p*-value (e.g., Bonferroni) since the latter methodology has its own limitations [31]. The sample size of 162 allows examination of associations that have a medium effect size in the terminology of Cohen [32].

## 3. Results

### 3.1. Study Population

The population consisted of 162 individuals with a mean age of 59 ± 14 years, 125 (77.2%) self-identified as male, 101 (62.3%) as White, and 51 (31.5%) as Hispanic (Table 1).

78.4% of the population (127 individuals) reported mild or greater DED symptoms with a DEQ5 score ≥ 6. A substantial proportion of participants had co-morbid cardiovascular, autoimmune, and endocrine conditions, which were mostly similar across the two groups (Table 1). Individuals who underwent objective autonomic testing and those who did not (based on individual availability for each test) had similar baseline characteristics (Supplementary Table S1). Trends in SBP, DBP, PP, and HR during orthostatic challenge are depicted for the population (Supplementary Figure S1). Overall, SBP, DBP, and HR increased from the supine baseline to the standing position, whereas PP decreased.

### 3.2. Relationships Between DED and Autonomic Metrics

Ocular symptoms were related to autonomic symptoms. COMPASS-31 total and subscores (except for bladder function) were significantly different between the DED and non-DED symptom groups (Table 2).

All ocular symptoms were positively correlated with multiple autonomic domains. The strongest associations were between (1) OSDI and pupillomotor scores (r = 0.67, *p* < 0.001), and (2) DEQ5 and secretomotor (r = 0.63, *p* < 0.001) and pupillomotor (r = 0.65, *p* < 0.001) scores (Table 3). Associations were slightly attenuated but remained statistically significant in sensitivity analyses excluding ocular-related COMPASS-31 items (Supplementary Table S2).

**Figure 1:**
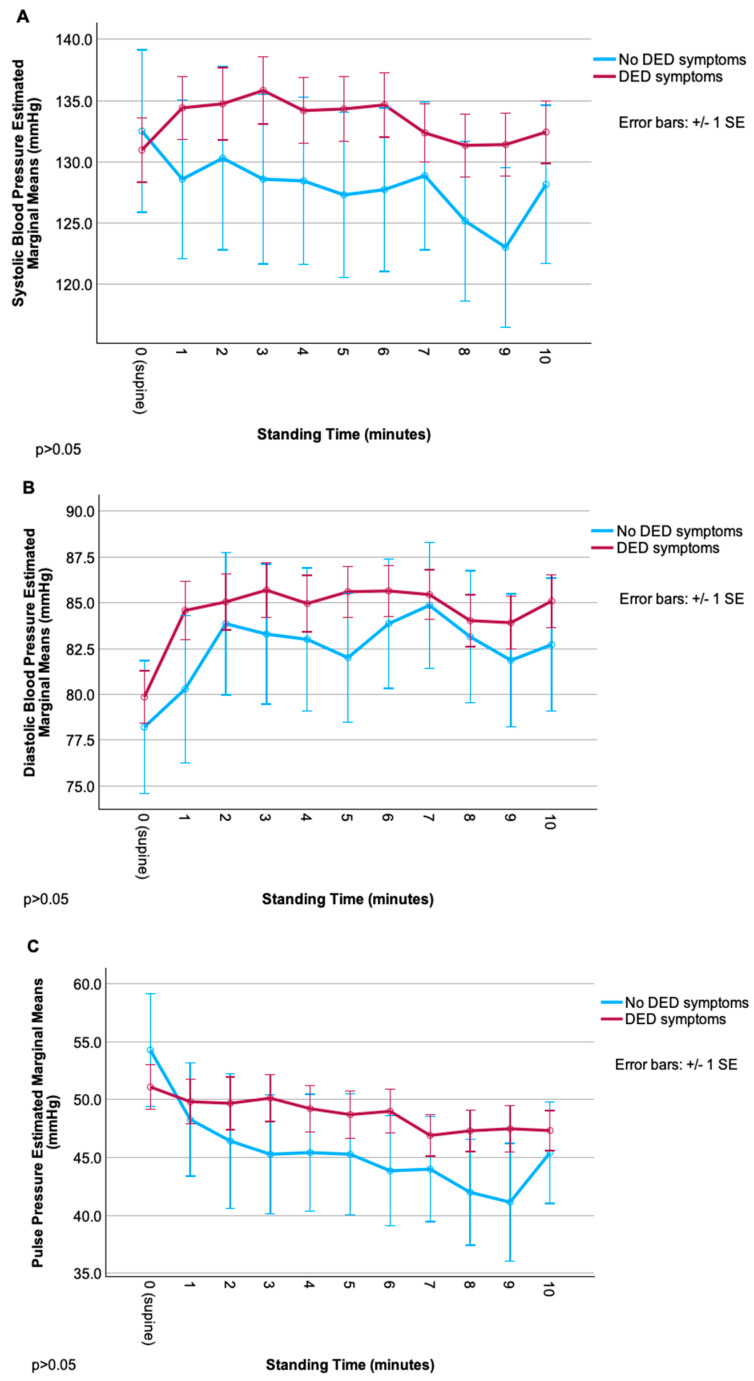

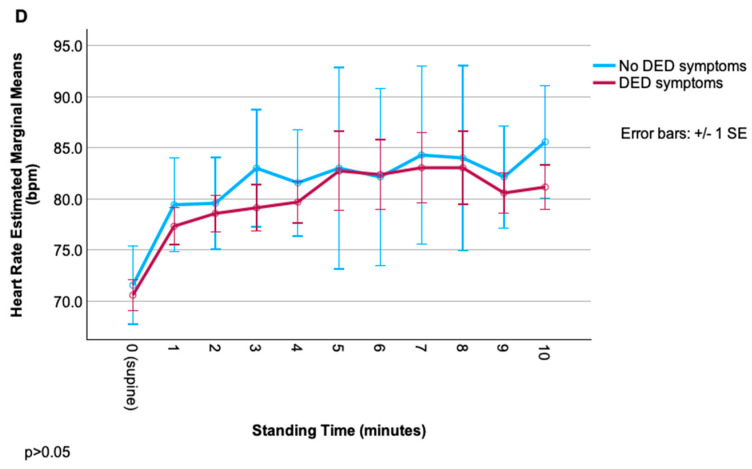
Change in cardiovascular parameters (systolic blood pressure, diastolic blood pressure, pulse pressure, heart rate) during the NASA lean test, grouped by dry-eye disease (DED) symptom status. (**A**) Systolic blood pressure over time in DED vs. non-DED symptom groups. (**B**) Diastolic blood pressure over time in DED vs. non-DED symptom groups. (**C**) Pulse pressure over time in DED vs. non-DED symptom groups. (**D**) Heart rate over time in DED vs. non-DED symptom groups.

Autonomic signs did not differ significantly by DED symptom status, including frequencies of OH and POTS, HRV metrics (Table 2), and changes in SBP, DBP, PP, and HR (Figure 1) over time.

Though trajectories of each measure appeared different between groups (especially for SBP, with an overall increase from baseline in DED vs. an overall decrease in the non-DED group), repeated-measures ANOVA revealed no significant main effects of group, time, or their interaction (all *p* > 0.05), likely due to high within-group variability. Relationships were noted between ocular symptom severity and cardiovascular autonomic parameters, but these correlations were less robust than for autonomic symptoms. Overall, pain symptoms (including neuropathic symptoms) were more related to signs of autonomic dysfunction than composite DED symptoms. Of these, relationships were strongest for rating of evoked pain to hot/cold and various autonomic signs including (1) difference in SBP (r = −0.44, *p* < 0.01) and DBP (r = −0.48, *p* < 0.01) from baseline to 8 min; (2) change in HR from baseline to 4 (r = 0.36), 6 (r = 0.39), and 7 (r = 0.38) minutes (*p* < 0.01 for all); and (3) HRV metrics: r = 0.41 and r = 0.43 for SDNN and RMSSD, respectively (*p* < 0.05 for both) (Table 3).

### 3.3. Multivariable Models

Forward stepwise regression analyses controlling for demographics (age, gender, ethnicity, and race), medical comorbidities, ocular comorbidities, and medication listed in Table 1 were performed with the dependent variables being ocular symptoms and the independent variables being autonomic symptoms and signs. To maximize sample size (N = 50), the reported models focused on autonomic symptom domains and orthostatic variables, as fewer individuals underwent HRV testing. Across all models, secretomotor and pupillomotor symptoms remained associated with ocular symptoms. Sensitivity analyses retained associations with the modified secretomotor domain, as well as with orthostatic and gastrointestinal domains (Supplementary Table S3). The autonomic signs most related to ocular symptoms were changes in SBP (2 min-baseline, 4 min-baseline, 7 min-baseline, and 8 min-baseline, which were most closely related to neuropathic symptoms (pain with wind, pain with light, total neuropathic pain score) and current ocular pain intensity rating. Changes in DBP and PP at various time points were also related to symptoms, namely neuropathic symptoms (pain with light, pain with hot/cold) and ocular pain intensity ratings (current, worst pain over the past week). Changes in HR were variably linked to both composite DED symptoms (OSDI) and neuropathic (pain with wind and hot/cold) symptoms (Table 4, Supplementary Table S4). In a model including all predictors (autonomic symptoms, orthostatic metrics, HRV metrics; N = 24), the Lf/Hf ratio was the metric most closely related to current ocular pain.

## 4. Discussion

When investigating relationships between autonomic metrics and ocular symptoms in a long-COVID population, ocular symptom severity was found to be related to all autonomic symptom domains, except for bladder function. Of all domains, secretomotor and pupillomotor symptoms were most closely related to composite DED symptoms (OSDI) and ocular pain intensity (NRS and NPSI-Eye). Though considerable overlap exists between eye-related items on the COMPASS-31 and the ocular questionnaires, sensitivity analyses revealed persistent associations with multiple autonomic domains. In contrast, autonomic signs were less strongly related to ocular symptoms, but with some modest correlations noted, in particular with respect to ocular pain features. Specifically, two minutes after standing, smaller increases in SBP were associated with pain evoked by light. During the intermediate phase of standing (4–7 min), this relationship reversed, with greater SBP increases linked to pain evoked by light and wind. By the final phase of standing (8 min), the association again shifted, with smaller SBP increases corresponding to current pain intensity and neuropathic pain descriptors. Fluctuations in relationships across time points were also noted with respect to DBP and PP, and in more restrictive models that included HRV, a positive association between the Lf/Hf ratio and ocular pain was noted. Because ocular symptoms were queried once and not at the time of autonomic testing, these findings do not permit temporal or causal inference and are physiologically difficult to interpret. Orthostatic BP and HR responses are also influenced by various situational factors and, in isolation, cannot be interpreted as evidence of objective autonomic dysfunction. Still, the observed pattern suggests that individuals with greater ocular pain may demonstrate variations in autonomic regulation.

The present findings share similarities with prior reports that examined associations in diseases relevant to DED. In a British study, participants from the UK Primary Sjögren’s Syndrome Registry (UKPSSR) were matched by age to community controls, and a longer version of the COMPASS questionnaire (73-items with ten domains) captured dysautonomia symptoms. Sjögrens disease (SjD) cases had higher COMPASS scores than controls (median (interquartile range [IQR]) 35.5 (20.9–46) vs. 14.8 (4.4–30.2), *p* < 0.01), with higher scores in seven of the ten domains [33]. Furthermore, correlations were noted between DED and dysautonomia symptoms in SjD cases, with “dryness” (0–10) intensity related to the COMPASS score (R^2^ = 0.14), an association that persisted in multivariable models. Compared to global dryness, global pain scores (0–10) were more closely associated with dysautonomia symptoms (R^2^ = 0.26), which complements the present findings that ocular dryness and pain were both related to dysautonomia symptoms, but only ocular pain to dysautonomia signs. Similar to the findings herein, an Italian study of 19 individuals with SjD and 17 controls reported significantly higher total COMPASS-31 scores (27.0 ± 10.9 vs. 9.0 ± 5.7; *p* < 0.0001). Among the subdomains, secretomotor (3.2 ± 1.5 vs. 0.0 ± 0.0; *p* < 0.0001) and pupillomotor (7.9 ± 2.9 vs. 2.4 ± 2.9; *p* = 0.0003) scores were most strongly associated with SjD [34]. Collectively, these data highlight associations between ocular and autonomic symptoms across different populations.

Signs of dysautonomia have also been investigated in sicca populations, with studies reporting inconsistent associations with ocular symptoms and generally no associations with ocular signs. For example, in a UK study of 27 individuals with SjD, no relationship was found between cardiovascular reflex indices (HRV, BP, and HR measured supine and standing) and tear production assessed by Schirmer’s test [35]. Similarly, a Swedish study reported no differences in orthostatic SBP and DBP ratios between SjD patients with normal versus abnormal Schirmer’s test (≤10 mm/5 min) and staining scores (≥8) [36]. Similar findings have been reported in Asia, with a Korean study stratifying 154 individuals with SjD based on HRV results (SDNN < 30 ms in patients < 50 years and SDNN < 20 ms in patients ≥ 50 years versus higher values in each group). Ocular symptoms (OSDI and the EULAR Sjögren’s Syndrome Patient Reported Index [ESSPRI] dryness sub-score) and signs (staining, tear film break-up time, and Meibomian gland dysfunction grade) did not differ across ANS groups. Notably, 42% of SjD cases showed reduced parasympathetic activity (Lf/Hf ratio > 1.6), yet DED signs and symptoms remained similar between those with and without parasympathetic dysfunction [37]. Other studies have used autonomic tests not included in the present study and reported similar findings. For example, in one study of 41 individuals with SjD, those with parasympathetic dysfunction (defined as a Valsalva ratio < 1.2 or a maximum–minimum HR during forced respiration < 15 bpm) showed similar DED signs compared with those without dysfunction [38]. However, some associations were noted between autonomic dysfunction signs and symptoms. In one UK study of individuals with SjD, dysautonomia was assessed using beat-to-beat HRV during 10 min of supine rest, as well as changes in SBP and DBP with orthostasis and Valsalva and ocular symptoms (EULAR sicca score) and signs (Schirmer’s test) were measured. The sicca score correlated with the Lf/Hf ratio (R^2^ = 0.3, *p* = 0.02) and DBP variability (R^2^ = 0.4, *p* = 0.003), whereas no associations were found with ocular signs [39]. Taken together, the findings herein and prior studies highlight inconsistent associations between autonomic measures and DED symptoms, and predominantly negative results with respect to DED signs.

The contribution of autonomic dysfunction to ocular symptoms in long-COVID remains uncertain, as multiple mechanisms could underlie these symptoms. Acutely, COVID-19 infection may impair tear stability and production, but whether these abnormalities persist chronically is uncertain [40]. Moreover, tear changes alone are unlikely to account for symptoms, given the well-established disconnect between ocular symptoms and DED signs [41]. Ocular surface inflammation has also been described during acute infection, with elevations of interferon-γ, tumor necrosis factor-α, interleukin-5, interleukin-8, and granulocyte-monocyte colony-stimulating factor in the tears of individuals with positive conjunctival SARS-CoV-2 swabs, but whether a pro-inflammatory ocular surface state endures is unknown [42,43]. Another potential mechanism is corneal nerve injury: studies using in vivo confocal microscopy in long-COVID patients have demonstrated reduced nerve density, shorter and more tortuous nerve fibers, and increased dendritic cell infiltration compared with controls [44,45,46]. At a central level, central sensitization—amplified neural signaling within the CNS resulting in pain hypersensitivity—may underlie ocular symptoms [47,48]. In fact, COVID-19 can promote neuroinflammation, releasing inflammatory mediators in the CNS that may drive chronic pain across multiple body sites [49,50]. The present study adds to this literature and raises the possibility that autonomic instability may co-occur with ocular symptoms in long-COVID, though causal or mechanistic relationships cannot be extrapolated from the present data.

Dysautonomia in long-COVID may arise through several mechanisms. The following hypotheses derived from prior literature are presented here as conceptual frameworks rather than explanations directly supported by the present data. During acute infection, the virus may invade somatosensory nerves and subsequently reach the autonomic nervous system, potentially spreading to the vagal nuclei [51,52]. Alternatively, COVID-19 may directly invade the autonomic nervous system at the level of the sympathetic chain. In one study, SARS-CoV-2 was detected in the superior cervical ganglia (SCG) of transgenic hACE2 mice inoculated with the virus, with viral RNA present in 97% of SCG neurons and associated with marked pathology, including vacuolation and loss of normal ganglionic architecture [52]. In either case, the virus is capable of reaching the autonomic nervous system, as shown by detection of SARS-CoV-2 RNA in the vagus nerves of 23 individuals who died from COVID-19, where viral load correlated positively with complement activation along nerve axons [53]. Interestingly, viral behavior may differ between the PNS and ANS, with viral release observed from PNS but not ANS neurons, and more severe cytopathology occurring in ANS neurons [52]. It remains unclear whether the link between long-COVID and dysautonomia is driven by persistent virus within autonomic ganglia, by structural nerve damage from infection, or by virus-induced autoimmunity contributing to ongoing symptoms and signs [54]. Evidence supporting the latter hypothesis includes high serum levels of G-protein coupled receptor functional autoantibodies (GPCR-fAABs) in individuals with long-COVID, including specific combinations of GPCR-fAABs implicated in autonomic conditions (e.g., POTS) [55]. These potential mechanisms of ANS dysfunction—whether it be viral persistence, structural injury, and/or autoimmunity—may ultimately have an impact at the level of the ocular surface, contributing to persistent symptoms in long-COVID patients.

As with all studies, interpretation of these results must be considered within the context of the study’s limitations. First, the cross-sectional study design cannot support conclusions about directionality. Because autonomic function testing was not performed concurrently with ocular symptom questionnaires, the observed correlations cannot be interpreted temporally or mechanistically. Second, statistical power and generalizability are constrained by the small sample size that underwent autonomic testing. Within the sample that did undergo orthostatic testing, several patients were unable to complete the 10-min NASA lean test (primarily due to musculoskeletal pain with prolonged standing), leading to attenuation of data at later time points and inconsistencies in sample size during analysis. Because the cohort that underwent objective testing was much smaller than the cohort with subjective symptom data, differences in correlation magnitude between objective and subjective data cannot be interpreted conclusively. Although baseline characteristics were compared between patients who underwent testing and those who did not, unmeasured differences may remain, and findings may not generalize to the entire cohort. Forward stepwise regression models performed with N ≈ 50 raise concern for overfitting, but models contained 2–6 predictors, yielding subjects-per-variable (SPV) of 7.5–25, all greater than the threshold of 2 SPV considered adequate for estimation of linear regression coefficients [56]. Third, the pupillomotor and secretomotor domains of the COMPASS-31 contain items directly related to eye dryness, visual discomfort, and light sensitivity. Therefore, correlations between these domains may be partially attributed to shared content rather than true autonomic dysfunction. Still, correlations are reported with other autonomic domains, some of which persist in multivariable models (e.g., vasomotor), which suggests that item overlap does not account for the overall spectrum of findings. Furthermore, sensitivity analyses excluding the pupillomotor domain and the ocular dryness item of the secretomotor domain retain many significant associations. Fourth, signs of tear dysfunction were not assessed; therefore, findings relate only to ocular symptom burden rather than structural or tear film abnormalities. This prevented assessment of relationships between autonomic metrics and other aspects of DED, and likewise, other aspects of long-COVID (viral serologies, immune parameters) were not available. Fifth, the study cohort primarily comprised older male veterans, which may limit generalizability. Autonomic response to orthostasis has been shown to differ by gender [57], and the incidence of DED and ocular pain is known to be higher in women [58]. Similarly, veterans and non-veterans may differ in comorbidities, environmental exposures, and healthcare utilization, and the severity of long-COVID symptoms, autonomic profiles, and ocular symptoms may differ between younger and older patient populations. Finally, based on the results of the autonomic testing, it cannot be further determined which aspects of the autonomic system were dysfunctional (sympathetic versus parasympathetic). Nonetheless, this study had strengths in that it examined both symptoms and signs of dysautonomia as they related to ocular symptoms.

## 5. Conclusions

In conclusion, this study highlights associations between ocular symptoms and dysautonomia in the long-COVID population that warrant further investigation. Strong relationships were observed between ocular symptoms, notably ocular pain and neuropathic ocular pain, and dysautonomia symptoms, most robustly pupillomotor and secretomotor. In addition, ocular pain ratings and neuropathic pain symptoms were associated with modest changes in PP, DBP, and SBP during orthostatic challenge and with the Lf/Hf ratio in secondary models, suggesting that ocular pain may co-occur with variations in autonomic activity. Future studies are needed to continue this work and investigate the contribution of dysautonomia signs (tested using various methodologies) on an expanded list of ocular investigations, including a comprehensive assessment of tear film and ocular surface parameters, corneal sensitivity, and corneal nerve status. An expanded understanding of the connection between the eye and the ANS can allow for better diagnostics and potentially targeted therapeutics, with the goal of developing a multidisciplinary approach to long-COVID management. Various neuromodulatory therapies have been proposed for autonomic dysfunction, with vagal nerve stimulation and transcutaneous electrical nerve stimulation specifically explored in the context of long-COVID [59,60], as well as for chronic neuropathic ocular pain [61,62]. Further research is needed to clarify how targeting autonomic dysfunction influences ocular symptoms, and vice versa, with neuromodulation as a possible avenue for this. This line of investigation is needed as intervening on both fronts concurrently may substantially improve function and enhance patients’ quality of life.

On a broader scale, these findings reinforce long-COVID as a heterogeneous, multisystem condition for which uniform diagnostic guidelines remain limited. Identifying ocular pain in a subset of long-COVID patients and its association with markers of autonomic dysfunction may support earlier detection and improved characterization of disease heterogeneity. Recognition that long-COVID spans multiple organ systems—including the eye—highlights the need for coordinated, multidisciplinary care, which is often lacking in current fragmented healthcare models [63]. Early diagnosis is critical, as delayed recognition can adversely affect quality of life, mental health, social functioning, and economic productivity at individual and societal levels [64]. As understanding of dysautonomia and ocular involvement in long-COVID advances, integrated multispecialty care models will be essential to overcome existing barriers to diagnosis and management.

## Figures and Tables

**Table 1:** Demographics, comorbidities, and medications, grouped by dry-eye disease (DED) symptom status.

	No DED Symptoms (DEQ5 < 6) (n = 35)	DED Symptoms (DEQ5 ≥ 6) (n = 127)	*p*-Value
Demographics			
Age (mean ± SD)	58.86 ± 16.91	59.39 ± 12.65	0.86
Gender, male, % (n)	85.7% (30)	74.8% (95)	0.17
Race, white, % (n)	28.1% (9)	30.3% (36)	0.82
Ethnicity, Hispanic, % (n)	21.9% (7)	35.2% (44)	0.15
Medical Comorbidities, % (n)			
Hypertension	57.1% (20)	58.3% (74)	0.91
Coronary artery disease	17.1% (6)	11.8% (15)	0.41
Congestive heart failure	14.3% (5)	7.1% (9)	0.18
Type II diabetes	25.7% (9)	20.5% (26)	0.51
Thyroid disease	5.7% (2)	9.4% (12)	0.49
Obstructive sleep apnea	48.6% (17)	55.9% (71)	0.44
Chronic lung disease (COPD, asthma, ILD)	20% (7)	20.5% (26)	0.95
Depression	34.3% (12)	48.8% (62)	0.13
Anxiety	22.9% (8)	40.2% (51)	0.06
Vitamin B12 deficiency	14.3% (5)	21.3% (27)	0.36
BPH	**8.6% (3)**	**27.6% (35)**	**0.02**
Alcohol use disorder	5.7% (2)	11.8% (15)	0.29
Chronic liver disease	**5.7% (2)**	**20.5% (26)**	**0.04**
HIV/AIDS	2.9% (1)	1.6% (2)	0.62
Rheumatoid arthritis	0% (0)	2.4% (3)	0.36
Sjögrens disease	0% (0)	3.1% (4)	0.29
Ocular comorbidities, % (n)			
Cataract surgery	8.6% (3)	15% (19)	0.33
Refractive surgery	8.6% (3)	7.1% (9)	0.77
Oral medications, % (n)			
Diuretic	28.6% (10)	25.2% (32)	0.69
Beta blocker	**48.6% (17)**	**28.3% (36)**	**0.02**
Alpha blocker	22.9% (8)	30.7% (39)	0.37
Antidepressant	25.7% (9)	43.3% (55)	0.06
Anti-anxiety	22.9% (8)	27.6% (35)	0.58
NSAID	51.4% (18)	44.9% (57)	0.49

Bold = variable significantly different (*p* < 0.05) between the groups. COPD = chronic obstructive pulmonary disease, ILD = interstitial lung disease, BPH = benign prostatic hyperplasia, HIV/AIDS = human immunodeficiency virus/acquired immunodeficiency syndrome, NSAID = non-steroidal anti-inflammatory drug.

**Table 2:** Symptoms and signs of dysautonomia, grouped by dry-eye disease (DED) symptom status.

	No DED Symptoms (DEQ5 < 6)	DED Symptoms (DEQ5 ≥ 6)	*p*-Value
Dysautonomia symptoms (mean ± SD)	n = 35	n = 127	
COMPASS-31 total	**20.92 ± 15.38**	**37.71 ± 18.05**	**<0.001**
COMPASS-31 subscores			
Orthostatic	**11.54 ± 10.923**	**17.49 ± 11.09**	**0.003**
Vasomotor	**0 ± 0**	**0.83 ± 1.34**	**<0.001**
Secretomotor	**2.52 ± 3.19**	**6.19 ± 3.63**	**<0.001**
Gastrointestinal	**4.50 ± 3.88**	**8.66 ± 4.63**	**<0.001**
Bladder	1.24 ± 2.61	1.71 ± 2.28	0.15
Pupillomotor	**1.11 ± 1.05**	**2.83 ± 1.19**	**<0.001**
Dysautonomia signs			
Orthostatic intolerance, % (n)	n = 9	n = 54	
SBP OH	0% (0)	0% (0)	1.00
DBP OH	0% (0)	1.9% (1)	0.68
POTS at any time point	0% (0)	9.3% (5)	0.34
Heart-rate variability, mean ± SD	n = 3	n = 35	
SDNN	44.6 ± 26.63	54.14 ± 30.62	0.61
RMSSD	60.9 ± 54.45	52.83 ± 35.12	0.72
Lf/Hf ratio	0.83 ± 0.51	3.46 ± 8.56	0.60

Bold = variable significantly different (*p* < 0.05) between the groups. COMPASS-31 = 31 Item Composite Autonomic Symptom Score, OH = orthostatic hypotension, POTS = postural orthostatic tachycardia, SBP = systolic blood pressure, DBP = diastolic blood pressure, RMSSD = root mean square of successive differences between normal heartbeats, SDNN = standard deviation of interbeat intervals of normal sinus beats, Lf/Hf = ratio of low-frequency to high-frequency power bands of heart-rate oscillations.

**Table 3:** Pearson correlations (r) between ocular surface symptoms and dysautonomia symptoms and signs.

	DEQ5	OSDI	NRS-1	NRS-2	NRS-3	NRS-4	NPSI-Eye 1	NPSI-Eye 2	NPSI-Eye 3	NPSI-Eye 4	NPSI-Eye Total
Autonomic symptoms											
Orthostatic	**0.31**	**0.40**	**0.27**	**0.30**	**0.25**	**0.17**	**0.24**	**0.30**	**0.30**	**0.19**	**0.36**
Vasomotor	**0.33**	**0.36**	**0.24**	**0.25**	**0.32**	**0.22**	**0.31**	**0.21**	**0.30**	**0.33**	**0.36**
Secreto motor	**0.63**	**0.56**	**0.45**	**0.46**	**0.43**	**0.50**	**0.37**	**0.49**	**0.43**	**0.40**	**0.58**
GI	**0.36**	**0.48**	**0.38**	**0.34**	**0.30**	**0.23**	**0.27**	**0.33**	**0.31**	**0.31**	**0.36**
Bladder	0.10	**0.23**	0.08	0.07	0.03	0.06	**0.19**	0.12	0.162	0.08	0.14
Pupillo motor	**0.65**	**0.67**	**0.51**	**0.54**	**0.43**	**0.43**	**0.32**	**0.36**	**0.55**	**0.30**	**0.51**
Total	**0.49**	**0.58**	**0.41**	**0.43**	**0.37**	**0.32**	**0.36**	**0.42**	**0.42**	**0.32**	**0.50**
Autonomic signs											
ΔSBP_2min-baseline_	0.22	0.13	−0.06	−0.02	−0.12	0.07	0.09	−0.04	−0.12	−0.18	−0.09
ΔSBP_3min-baseline_	0.17	0.15	0.02	0.001	−0.01	−0.01	−0.02	0.101	0.06	−0.20	−0.02
ΔSBP_4min-baseline_	0.15	0.06	0.07	0.02	0.06	0.09	0.15	0.07	0.26	0.05	0.20
ΔSBP_5min-baseline_	0.21	0.15	0.04	−0.06	−0.04	0.03	−0.11	0.09	0.08	−0.19	−0.04
ΔSBP_6min-baseline_	0.16	0.09	−0.06	−0.14	−0.16	0.08	−0.02	−0.01	0.03	−0.22	−0.05
ΔSBP_7min-baseline_	−0.06	−0.11	−0.26	−0.24	−0.26	−0.25	−0.24	−0.05	−0.16	−0.19	−0.25
ΔSBP_8min-baseline_	0.05	−0.08	−0.25	−0.19	−0.25	−0.13	−0.18	−0.24	−0.23	**−0.44**	**−0.36**
ΔSBP_9min-baseline_	0.05	−0.03	−0.09	−0.09	−0.14	−0.15	−0.04	0	−0.03	−0.09	−0.08
ΔSBP_10min-baseline_	0.04	0.04	−0.17	−0.14	−0.18	−0.09	−0.09	−0.01	−0.09	−0.09	−0.10
ΔDBP_2min-baseline_	0.001	−0.04	−0.07	−0.11	−0.19	−0.11	−0.12	0.01	−0.12	−0.18	−0.12
ΔDBP_3min-baseline_	−0.01	−0.01	−0.09	−0.09	−0.11	−0.18	−0.19	0.11	−0.02	−0.23	−0.09
ΔDBP_4min-baseline_	0.09	−0.08	−0.08	−0.12	−0.18	−0.07	−0.05	−0.07	−0.02	−0.26	−0.12
ΔDBP_5min-baseline_	0.03	−0.05	−0.18	−0.19	−0.21	−0.17	−0.26	0.03	−0.05	−0.21	−0.17
ΔDBP_6min-baseline_	0.05	0.02	−0.09	−0.16	−0.19	−0.002	−0.11	−0.01	−0.10	−0.16	−0.12
ΔDBP_7min-baseline_	−0.09	−0.09	**−0.28**	**−0.34**	**−0.35**	−0.18	**−0.30**	−0.03	−0.24	−0.26	**−0.28**
ΔDBP_8min-baseline_	−0.03	−0.11	−0.23	−0.23	**−0.30**	−0.19	**−0.28**	−0.21	**−0.32**	**−0.48**	**−0.41**
ΔDBP_9min-baseline_	−0.04	−0.09	−0.13	−0.16	−0.25	−0.13	−0.19	0.08	−0.21	−0.08	−0.16
ΔDBP_10min-baseline_	−0.003	0.01	−0.07	−0.12	−0.16	−0.16	−0.10	0.07	−0.11	−0.09	−0.07
ΔPP_2min-baseline_	0.24	0.19	0.02	0.07	0.04	0.09	0.18	−0.06	0.01	−0.01	−0.01
ΔPP_3min-baseline_	0.21	0.19	0.08	0.05	0.05	0.10	0.09	0.06	0.09	−0.09	0.04
ΔPP_4min-baseline_	0.22	**0.29**	0.05	0.03	0.10	0.11	0.001	0.09	0.08	0.14	0.09
ΔPP_5min-baseline_	0.24	0.21	0.17	0.06	0.09	0.15	0.03	0.09	0.11	−0.11	0.05
ΔPP_6min-baseline_	0.17	0.09	−0.02	−0.06	−0.06	0.10	0.06	−0.004	0.10	−0.16	0.03
ΔPP_7min-baseline_	−0.01	−0.06	−0.12	−0.05	−0.07	−0.17	−0.08	−0.04	−0.03	−0.05	−0.10
ΔPP_8min-baseline_	−0.08	−0.02	−0.15	−0.09	−0.10	−0.03	−0.03	−0.17	−0.07	−0.23	−0.17
ΔPP_9min-baseline_	0.09	0.02	−0.03	−0.01	−0.02	−0.11	0.06	−0.05	0.09	−0.07	−0.003
ΔPP_10min-baseline_	0.09	−0.07	−0.10	−0.03	−0.07	−0.08	0.03	0.06	−0.05	**−0.29**	−0.08
ΔHR_2min-baseline_	−0.06	0.17	0.09	0.09	0.15	−0.05	−0.06	**0.39**	0.23	0.23	0.24
ΔHR_3min-baseline_	−0.06	0.25	0.09	0.04	0.15	0.05	−0.02	**0.29**	0.11	**0.33**	0.23
ΔHR_4min-baseline_	−0.07	0.22	0.03	0.06	0.12	0.04	−0.09	**0.32**	0.12	**0.36**	0.21
ΔHR_5min-baseline_	0.05	**0.33**	0.11	0.07	0.17	0.13	0.02	0.26	0.12	**0.34**	0.23
ΔHR_6min-baseline_	0.02	**0.35**	0.08	0.06	0.16	0.11	−0.003	0.27	0.08	**0.39**	0.22
ΔHR_7min-baseline_	0.01	**0.33**	0.09	0.06	0.17	0.09	−0.03	0.28	0.11	**0.38**	0.22
ΔHR_8min-baseline_	0.02	**0.32**	0.14	0.14	0.20	0.04	0.02	**0.30**	0.15	**0.36**	0.26
ΔHR_9min-baseline_	−0.03	0.11	0.05	0.09	0.12	−0.07	0.001	0.25	0.15	0.17	0.17
ΔHR_10min-baseline_	−0.15	0.17	0.02	0.01	0.12	−0.04	−0.08	**0.33**	0.087	**0.33**	0.17
HRV SDNN	−0.14	0.05	−0.03	0.03	0.17	0.30	−0.23	0.35	0.23	**0.41**	0.31
HRV RMSSD	0.05	0.07	0.02	0.02	0.12	0.30	−0.19	0.35	0.15	**0.43**	0.29
HRV Lf/Hf Ratio	−0.01	−0.16	−0.26	−0.07	0.07	−0.09	−0.18	0.02	0.02	−0.12	−0.05

Bold Green = significant positive correlation. Bold Red = significant negative correlation. DEQ5 = 5 Item Dry-Eye Questionnaire, OSDI = Ocular Surface Disease Index, NRS-1 = Numerical Rating Scale, average ocular pain over past week, NRS-2 = Numerical Rating Scale, worst ocular pain over past week, NRS-3 = Numerical Rating Scale, ocular pain now, NRS-4 = Numerical Rating Scale, ocular dryness now, NPSI-Eye 1 = 4-Question Neuropathic Pain Symptom Inventory modified for the Eye, burning pain, NPSI-Eye 2 = 4-Question Neuropathic Pain Symptom Inventory modified for the Eye, pain evoked by wind, NPSI-Eye 3 = 4-Question Neuropathic Pain Symptom Inventory modified for the Eye, pain evoked by light, NPSI-Eye 4 = 4-Question Neuropathic Pain Symptom Inventory modified for the Eye, pain evoked by hot/cold, NPSI-Eye Total = Total of 4-Question Neuropathic Pain Symptom Inventory modified for the Eye, ΔSBP = change in systolic blood pressure, ΔDBP = change in diastolic blood pressure, ΔPP = change in pulse pressure, ΔHR = change in heart rate, SDNN = standard deviation of interbeat intervals of normal sinus beats, RMSSD = root mean square of successive differences between normal heartbeats, Lf/Hf = ratio of low-frequency to high-frequency power bands of heart-rate oscillations.

**Table 4:** Forward stepwise linear regression analyses with ocular symptoms as dependent variables and autonomic symptoms and signs (orthostatic metrics) as independent variables, while controlling for demographics and comorbidities *.

Dependent Variable	N	Adjusted R^2^	Predictor	Standardized Beta	*p*-Value
DED symptoms					
DEQ5 (0–22)	50	0.51	Secretomotor	0.52	<0.001
			Pupillomotor	0.36	0.01
			ΔSBP_2min-baseline_	0.29	0.01
			Bladder	−0.35	0.003
OSDI (0–100)	50	0.56	Pupillomotor	0.68	<0.001
			ΔHR_5min-baseline_	0.26	0.01
Dryness right now (0–10)	50	0.37	Secretomotor	0.77	<0.001
			Gastrointestinal	−0.42	0.004
Ocular pain					
Average pain intensity over the past week (0–10)	50	0.40	Pupillomotor	0.53	<0.001
			ΔDBP_7min-baseline_	−0.29	0.01
Worst pain intensity over the past week (0–10)	50	0.53	Pupillomotor	0.45	<0.001
			Secretomotor	0.25	0.04
			Vasomotor	0.22	0.03
			ΔDBP_7min-baseline_	−0.24	0.02
Pain right now (0–10)	50	0.54	Secretomotor	0.46	<0.001
			Pupillomotor	0.25	0.03
			ΔSBP_8min-baseline_	−0.34	0.01
			ΔPP_5min-baseline_	0.25	0.04
Burning pain (0–10)	50	0.11	Secretomotor	0.36	0.01
Pain evoked by wind (0–10)	50	0.59	Pupillomotor	0.46	<0.001
			GI	0.36	0.001
			ΔSBP_7min-baseline_	0.36	0.02
			ΔPP_8min-baseline_	−0.47	0.002
			ΔHR_2min-baseline_	0.33	0.002
Pain evoked by light (0–10)	50	0.51	Pupillomotor	0.65	<0.001
			ΔSBP_4min-baseline_	0.39	<0.001
			ΔSBP_2min-baseline_	−0.32	0.01
Pain evoked by hot/cold (0–10)	45	0.67	Secretomotor	0.37	<0.001
			ΔDBP_9min-baseline_	0.28	0.01
			ΔPP_4min-baseline_	0.52	0.002
			ΔPP_6min-baseline_	−0.59	<0.001
			ΔHR_2min-baseline_	−0.38	0.002
			ΔHR_6min-baseline_	0.41	0.001
Total of 4 NPSI-Eye questions (0–40)	50	0.61	Secretomotor	0.40	<0.001
			Pupillomotor	0.35	0.002
			ΔSBP_4min-baseline_	0.28	0.01
			ΔSBP_8min-baseline_	−0.30	0.02

DEQ5 = 5 Item Dry-Eye Questionnaire, OSDI = Ocular Surface Disease Index, NPSI-Eye = 4-Question Neuropathic Pain Symptom Inventory modified for the Eye, ΔSBP = change in systolic blood pressure, ΔDBP = change in diastolic blood pressure, ΔPP = change in pulse pressure, ΔHR = change in heart rate, DED = dry-eye disease. * only significant relationships between ocular symptoms and autonomic metrics are reported.

## Data Availability

Data are available on request from the authors due to privacy reasons.

## Supplementary Materials

The following supporting information can be downloaded at: https://www.mdpi.com/article/10.3390/brainsci16020135/s1, Table S1: Demographics, comorbidities, and medications, grouped by objective autonomic testing status; Table S2: Pearson correlations (r) between ocular surface symptoms and dysautonomia symptoms after exclusion of ocular-related COMPASS-31 items; Table S3: Forward stepwise linear regression models with ocular symptoms as dependent variables and autonomic symptoms and signs (orthostatic metrics) as independent variables, after exclusion of ocular-related COMPASS-31 items and controlling for demographics and comorbidities; Table S4: Direction of Significant Associations Between NASA Lean Metrics and Dry-Eye Symptom Scores in Forward Regression Models; Figure S1: Change in cardiovascular parameters (systolic blood pressure, diastolic blood pressure, pulse pressure, heart rate) during the NASA lean test in all participants. A. Systolic blood pressure over time in all participants. B. Diastolic blood pressure over time in all participants. C. Pulse pressure over time in all participants. D. Heart rate over time in all participants; STROBE Statement—checklist of items that should be included in reports of observational studies.

## Abbreviations

The following abbreviations are used in this manuscript:

DED	Dry-eye disease
ANS	Autonomic nervous system
OH	Orthostatic hypotension
POTS	Postural orthostatic tachycardia syndrome
SBP	Systolic blood pressure
DBP	Diastolic blood pressure
PP	Pulse pressure
HR	Heart rate
HRV	Heart-rate variability
SDNN	Standard deviation of interbeat intervals of normal sinus beats
RMSSD	Root mean square of successive differences between normal heartbeats
Lf/Hf	Ratio of low-frequency to high-frequency power bands of heart-rate oscillations
COMPASS-31	31 Item Composite Autonomic Symptom Score
DEQ5	5 Item Dry-Eye Questionnaire
OSDI	Ocular Surface Disease Index
NRS	Numerical Rating Scale
NPSI-Eye	4-Question Neuropathic Pain Symptom Inventory modified for the Eye
