# Supplementary material for: Ocular Symptoms as a Marker of Dysautonomia in Long-COVID Patients: A Cross-Sectional Analysis
Source: Brain Sci. 2026 Jan 27;16(2):135. doi: 10.3390/brainsci16020135 (PMC12937984; doi:10.3390/brainsci16020135)
Supplement: Supplementary file 1 [file brainsci-16-00135-s001.zip › brainsci-4075148-supplementary.pdf]

Supplementary Material

Table S1. Demographics, comorbidities, and medications, grouped by objective autonomic testing status.

	**Autonomic testing not done (n=88)**	**Autonomic testing done (n=74)**	**P-value**
Demographics			
Age (mean±SD)	59.6±15.13	58.9±11.7	0.78
Gender, male, % (n)	72.7% (64)	82.4% (61)	0.14
Race, white, % (n)	**74.1% (60)**	**58.6% (41)**	**0.04**
Ethnicity, Hispanic, % (n)	29.4% (25)	36.1% (26)	0.37
Medical Comorbidities, % (n)			
Hypertension	58.0% (51)	58.1% (43)	0.98
Coronary artery disease	10.2% (9)	16.2% (12)	0.26
Congestive heart failure	6.8% (6)	10.8% (8)	0.37
Type II diabetes	21.6% (19)	21.6% (16)	0.99
Thyroid disease	6.8% (6)	10.8% (8)	0.37
Obstructive sleep apnea	52.3% (46)	56.8% (42)	0.57
Chronic lung disease (COPD, asthma, ILD)	18.2% (16)	23.0% (17)	0.45
Depression	38.6% (34)	54.1% (40)	0.05
Anxiety	31.8% (28)	41.9% (31)	0.18
Vitamin B12 deficiency	15.9% (14)	24.3% (18)	0.18
BPH	**15.9% (14)**	**32.4% (24)**	**0.01**
Alcohol use disorder	9.1% (8)	12.2% (9)	0.53
Chronic liver disease	15.9% (14)	18.9% (14)	0.61
HIV/AIDS	0% (0)	4.1% (3)	0.06
Rheumatoid arthritis	0% (0)	4.1% (3)	0.06
Sjögrens disease	3.4% (4)	1.4% (1)	0.40
Ocular comorbidities, % (n)			
Cataract surgery	12.5% (11)	14.9% (11)	0.66
Refractive surgery	5.7% (5)	9.5% (7)	0.36
Oral medications, % (n)			
Diuretic	25.0% (22)	27.0% (20)	0.77

Beta blocker	34.1% (30)	31.1% (23)	0.68
Alpha blocker	**21.6% (19)**	**37.8% (28)**	**0.02**
Antidepressant	**30.7% (27)**	**50.0% (37)**	**0.01**
Anti-anxiety	22.7% (20)	31.1% (23)	0.23
NSAID	**35.2% (31)**	**59.5% (44)**	**0.002**

Bold- variable significantly different (p<0.05) between the groups. BPH = benign prostatic hyperplasia, COPD = chronic obstructive pulmonary disease, ILD = interstitial lung disease, HIV/AIDS = human immunodeficiency virus/acquired immunodeficiency syndrome, NSAID = non-steroidal anti-inflammatory drug.

Table S2. Pearson correlations (r) between ocular surface symptoms and dysautonomia symptoms after exclusion of ocular-related COMPASS-31 items.

	DEQ5	OSDI	NRS-1	NRS-2	NRS-3	NRS-4	NPSI-Eye 1	NPSI-Eye 2	NPSI-Eye 3	NPSI-Eye 4	NPSI-Eye Total
Autonomic symptoms											
Orthostatic	**0.31**	**0.40**	**0.27**	**0.30**	**0.25**	**0.17**	**0.24**	**0.30**	**0.30**	**0.19**	**0.36**
Vasomotor	**0.33**	**0.36**	**0.24**	**0.25**	**0.32**	**0.22**	**0.31**	**0.21**	**0.30**	**0.33**	**0.36**
Secreto motor	**0.53**	**0.55**	**0.38**	**0.38**	**0.38**	**0.39**	**0.33**	**0.49**	**0.42**	**0.37**	**0.56**
GI	**0.36**	**0.48**	**0.38**	**0.34**	**0.30**	**0.23**	**0.27**	**0.33**	**0.31**	**0.31**	**0.36**
Bladder	0.10	**0.23**	0.08	0.07	0.03	0.06	**0.19**	0.12	0.16	0.08	0.14
Total	**0.42**	**0.53**	**0.36**	**0.38**	**0.34**	**0.26**	**0.32**	**0.41**	**0.39**	**0.31**	**0.45**

Bold Green=significant positive correlation. DEQ5 = 5 Item Dry Eye Questionnaire, OSDI = Ocular Surface Disease Index, NRS-1 = Numerical Rating Scale, average ocular pain over past week, NRS-2 = Numerical Rating Scale, worst ocular pain over past week, NRS-3 = Numerical Rating Scale, ocular pain now, NRS-4 = Numerical Rating Scale, ocular dryness now, NPSI-Eye 1 = 4-Question Neuropathic Pain Symptom Inventory modified for the Eye, burning pain, NPSI-Eye 2 = 4-Question Neuropathic Pain Symptom Inventory modified for the Eye, pain evoked by wind, NPSI-Eye 3 = 4-Question Neuropathic Pain Symptom Inventory modified for the Eye, pain evoked by light, NPSI-Eye 4 = 4-Question Neuropathic Pain Symptom Inventory modified for the Eye, pain evoked by hot/cold, NPSI-Eye Total = Total of 4-Question Neuropathic Pain Symptom Inventory modified for the Eye

Table S3. Forward stepwise linear regression models with ocular symptoms as dependent variables and autonomic symptoms and signs (orthostatic metrics) as independent variables, after exclusion of ocular-related COMPASS-31 items and controlling for demographics and comorbidities*.

**Dependent variable**	N	**Adjusted R^2**	**Predictor**	**Standardized beta**	**P-value**
DED symptoms					
DEQ5 (0-22)	45	0.58	Secretomotor	1.05	<0.001
			GI	-0.74	<0.001
			$\Delta PP_{5min\text{-}baseline}$	0.38	0.001
			$\Delta SBP_{4min\text{-}baseline}$	0.25	0.04
OSDI (0-100)	45	0.46	Secretomotor	0.51	<0.001
			$\Delta HR_{6min\text{-}baseline}$	0.31	0.01
Dryness right now (0-10)	45	0.29	Secretomotor	0.44	0.002
Ocular pain					
Average pain intensity over past week (0-10)	45	0.41	Secretomotor	0.43	<0.001
			$\Delta DBP_{7min\text{-}baseline}$	-0.41	0.002
Worst pain intensity over past week (0-10)	45	0.55	Secretomotor	0.51	<0.001
			$\Delta DBP_{7min\text{-}baseline}$	-0.59	<0.001
			$\Delta DBP_{4min\text{-}baseline}$	0.30	0.04
Pain right now (0-10)	45	0.55	Secretomotor	0.67	<0.001
			$\Delta SBP_{8min\text{-}baseline}$	-0.41	0.01
			$\Delta PP_{5min\text{-}baseline}$	0.85	0.001
			$\Delta DBP_{2min\text{-}baseline}$	0.53	0.002
			$\Delta SBP_{5min\text{-}baseline}$	-0.70	0.03
Pain evoked by wind (0-10)	45	0.54	Orthostatic	0.36	0.01
			GI	0.49	<0.001
Pain evoked by light (0-10)	45	0.30	Orthostatic	0.43	0.001
			$\Delta SBP_{4min\text{-}baseline}$	0.28	0.04
Pain evoked by hot/cold (0-10)	45	0.53	Secretomotor	0.33	0.004
			$\Delta DBP_{8min\text{-}baseline}$	-0.60	<0.001
			$\Delta DBP_{9min\text{-}baseline}$	0.28	0.03
			$\Delta HR_{6min\text{-}baseline}$	0.54	<0.001
			$\Delta HR_{2min\text{-}baseline}$	-0.29	0.03
	45	0.50	Orthostatic	0.31	0.02

Total of 4 NPSI-Eye questions (0-40)	Secretomotor	0.35	0.01
	$\Delta DBP_{8min\text{-}baseline}$	-0.45	0.002
	$\Delta DBP_{10min\text{-}baseline}$	0.30	0.03

DEQ5 = 5 Item Dry Eye Questionnaire, OSDI = Ocular Surface Disease Index, NPSI-Eye = 4-Question Neuropathic Pain Symptom Inventory modified for the Eye, ΔSBP = change in systolic blood pressure, ΔDBP = change in diastolic blood pressure, ΔPP = change in pulse pressure, ΔHR= change in heart rate, DED = dry eye disease. *only significant relationships between ocular symptoms and autonomic metrics reported.

Table S4. Direction of Significant Associations Between NASA Lean Metrics and Dry Eye Symptom Scores in Forward Regression Models

Time (min)	ΔSBP	ΔDBP	ΔPP	ΔHR
2	(+) DEQ5<br>(-) NPSI-Eye 3			(+) NSPI-Eye 2<br>(-) NPSI-Eye 4
3				
4	(+) NPSI-Eye 3<br>(+) NPSI-Eye total		(+) NPSI-Eye 4	
5			(+) NRS-3	(+) OSDI
6			(-) NPSI-Eye 4	(+) NPSI-Eye 4
7	(+) NPSI-Eye 2	(-) NRS-1<br>(-) NRS-2		
8	(-) NRS-3<br>(-) NPSI-Eye total	(-) NPSI-Eye 3	(-) NPSI-Eye 2	
9		(+) NPSI-Eye 4		
10				

(+) = positive beta coefficient
(-) = negative beta coefficient
Blank = no significant association
DEQ5 = 5 Item Dry Eye Questionnaire, OSDI = Ocular Surface Disease Index, NRS-1 = Numerical Rating Scale, average ocular pain over past week, NRS-2 = Numerical Rating Scale, worst ocular pain over past week, NRS-3 = Numerical Rating Scale, ocular pain now, NPSI-Eye 1 = Does your eye pain feel like burning?, NPSI-Eye 2 = Is your eye pain provoked or increased by wind?, NPSI-Eye 3 = Is your eye pain provoked or increased by light?, NPSI-Eye 4 = Is your eye pain provoked or increased by *contact* with something cold or hot (air conditioned/warm weather)?, NPSI-Eye Total = Total of 4-questions from Neuropathic Pain Symptom Inventory modified for the Eye, ΔSBP = change in systolic blood pressure, ΔDBP = change in diastolic blood pressure, ΔPP = change in pulse pressure, ΔHR= change in heart rate

Figure S1. Change in cardiovascular parameters (systolic blood pressure, diastolic blood pressure, pulse pressure, heart rate) during the NASA lean test in all participants. A. Systolic blood pressure over time in all participants. **B.** Diastolic blood pressure over time in all participants. **C.** Pulse pressure over time in all participants. **D.** Heart rate over time in all participants.

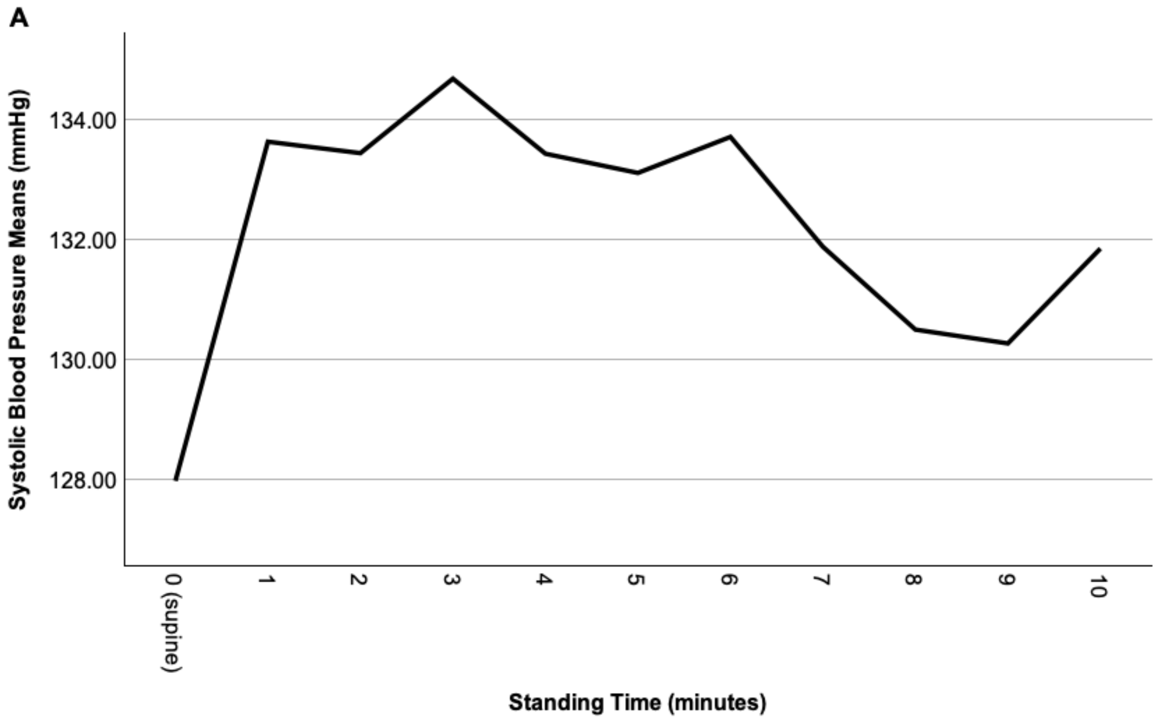

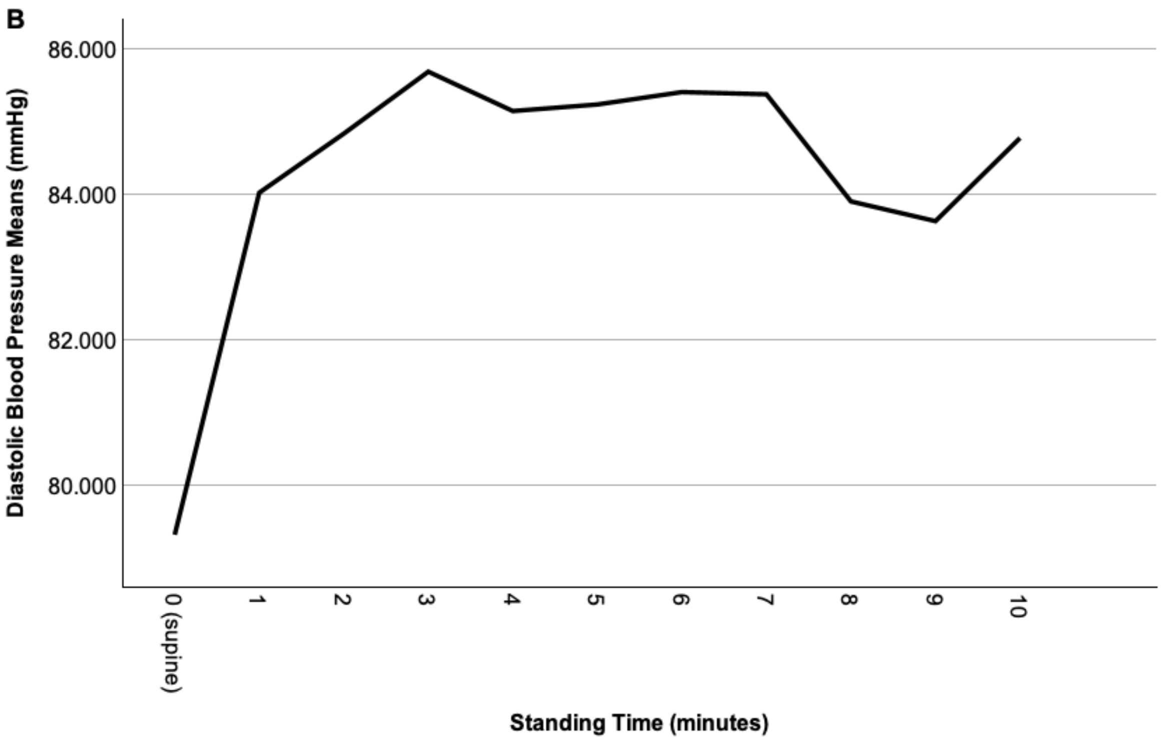
B
Diastolic Blood Pressure Means (mmHg)
86.000
84.000
82.000
80.000
0 (supine)
1
2
3
4
5
6
7
8
9
10
Standing Time (minutes)

C

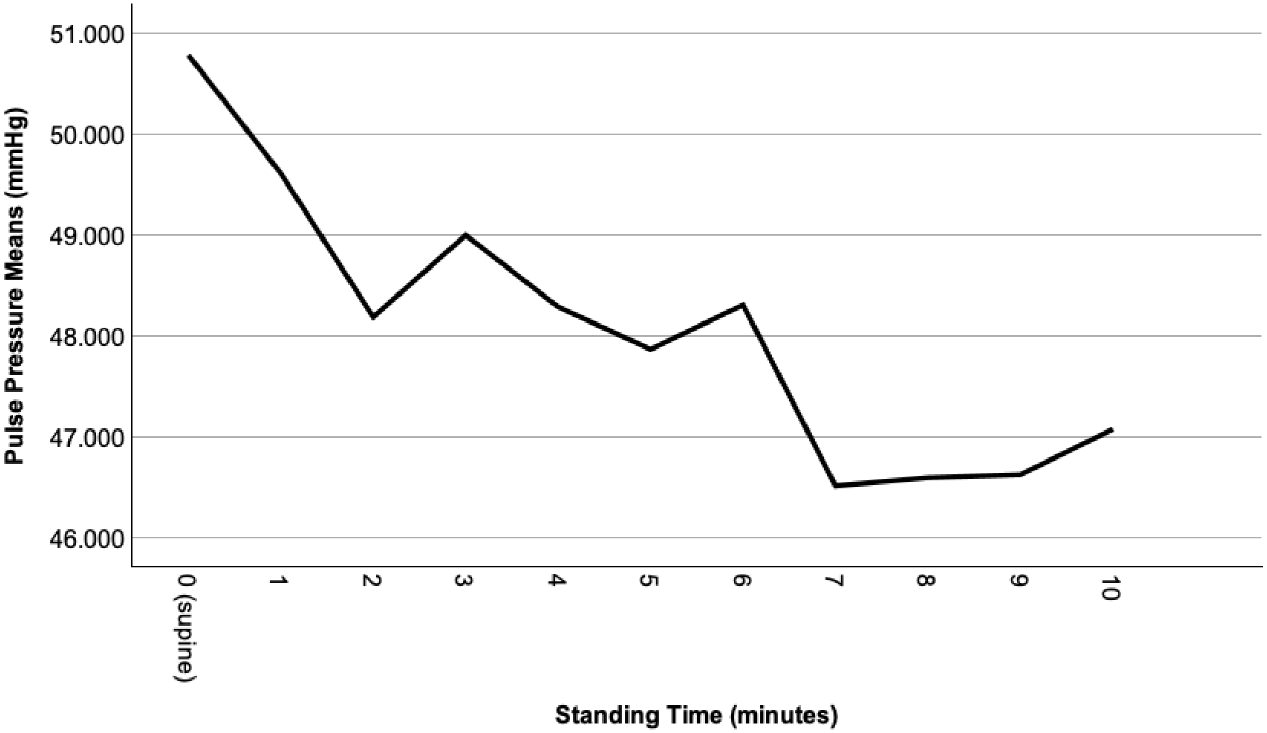
Pulse Pressure Means (mmHg)
51.000
50.000
49.000
48.000
47.000
46.000
0 (supine)
1
2
3
4
5
6
7
8
9
10
Standing Time (minutes)

D

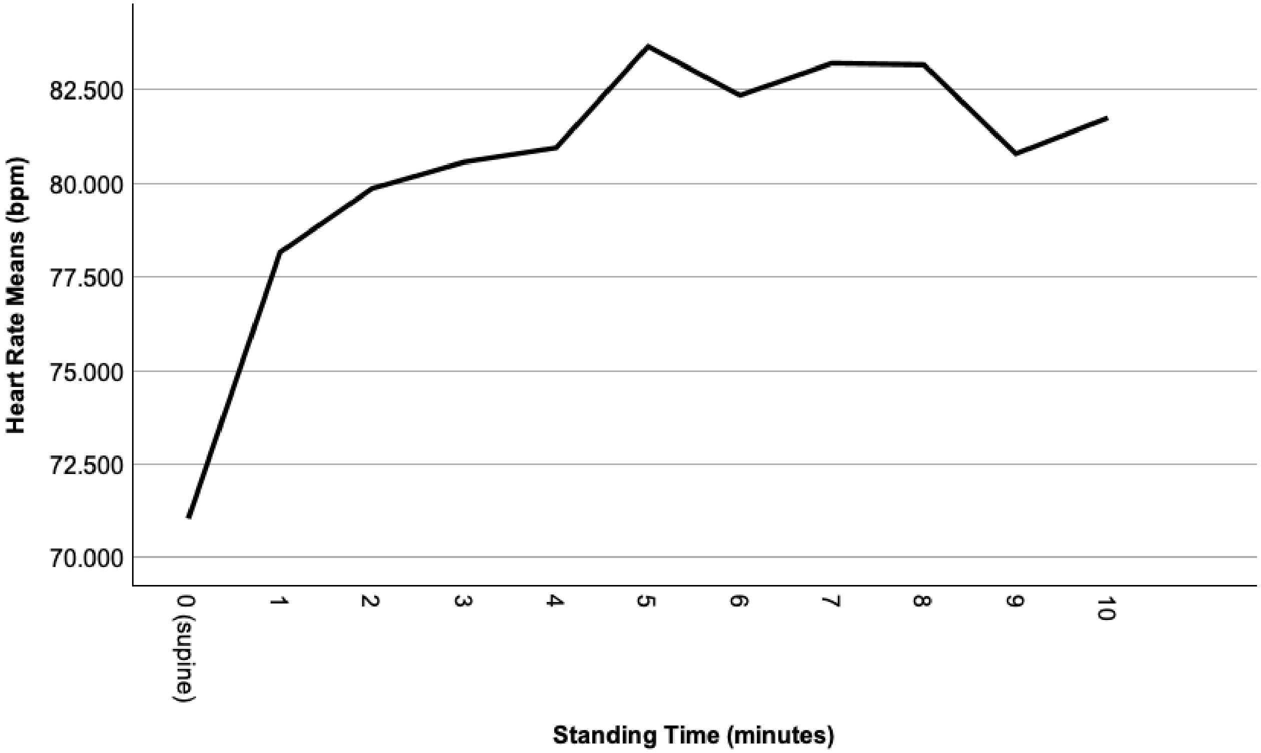

Heart Rate Means (bpm)
82.500
80.000
77.500
75.000
72.500
70.000
0 (supine)
1
2
3
4
5
6
7
8
9
10
Standing Time (minutes)

STROBE Statement—checklist of items that should be included in reports of observational studies

	Item No.	Recommendation	Page No.	Relevant text from manuscript
**Title and abstract**	1	(*a*) Indicate the study's design with a commonly used term in the title or the abstract	1	Title
		(*b*) Provide in the abstract an informative and balanced summary of what was done and what was found	1	Abstract (all text)
**Introduction**				
Background/rationale	2	Explain the scientific background and rationale for the investigation being reported	2	Introduction (paragraphs 1-3)
Objectives	3	State specific objectives, including any prespecified hypotheses	2	Introduction (paragraph 4)
**Methods**				
Study design	4	Present key elements of study design early in the paper	3	Materials and Methods (section 2.1)
Setting	5	Describe the setting, locations, and relevant dates, including periods of recruitment, exposure, follow-up, and data collection	3	Materials and Methods (section 2.1)
Participants	6	(*a*) *Cross-sectional study*—Give the eligibility criteria, and the sources and methods of selection of participants	3	Materials and Methods (section 2.1)
Variables	7	Clearly define all outcomes, exposures, predictors, potential confounders, and effect modifiers. Give diagnostic criteria, if applicable	3-4	Materials and Methods (sections 2.1, 2.3, 2.4, and 2.5)
Data sources/ measurement	8*	For each variable of interest, give sources of data and details of methods of assessment (measurement). Describe comparability of assessment methods if there is more than one group	3-4	Materials and Methods (sections 2.1-2.5)
Bias	9	Describe any efforts to address potential sources of bias	4	Materials and Methods (section 2.5)
Study size	10	Explain how the study size was arrived at	3	Materials and Methods (section 2.1)

Continued on next page

Quantitative variables	11	Explain how quantitative variables were handled in the analyses. If applicable, describe which groupings were chosen and why	4	Materials and Methods (sections 2.4-2.5)
Statistical methods	12	(*a*) Describe all statistical methods, including those used to control for confounding	4	Materials and Methods (section 2.5)
		(*b*) Describe any methods used to examine subgroups and interactions	4	Materials and Methods (section 2.5)
		(*c*) Explain how missing data were addressed	4	Materials and Methods (section 2.5)
		(*d*) *Cross-sectional study*—If applicable, describe analytical methods taking account of sampling strategy	N/A	N/A
		(e) Describe any sensitivity analyses	4	Materials and Methods (section 2.5)
**Results**				
Participants	13*	(a) Report numbers of individuals at each stage of study—eg numbers potentially eligible, examined for eligibility, confirmed eligible, included in the study, completing follow-up, and analysed	4-6	Results (sections 3.1-3.2; Tables 1-2)
		(b) Give reasons for non-participation at each stage	5	Results (section 3.1)
		(c) Consider use of a flow diagram	N/A	N/A (numbers of participants systematically reported in Tables 1-2)
Descriptive data	14*	(a) Give characteristics of study participants (eg demographic, clinical, social) and information on exposures and potential confounders	4-5	Results (section 3.1; Table 1)
		(b) Indicate number of participants with missing data for each variable of interest	4-6	Results (section 3.1; Tables 1-2)
Outcome data	15*	*Cross-sectional study*—Report numbers of outcome events or summary measures	4-6	Results (section 3.1, 3.2; Tables 1-2)
Main results	16	(*a*) Give unadjusted estimates and, if applicable, confounder-adjusted estimates and their precision (eg, 95% confidence interval). Make clear which confounders were adjusted for and why they were included	4-12	Results (section 3.1, 3.2, 3.3; Tables 1-4)
		(*b*) Report category boundaries when continuous variables were categorized	4-6	Results (section 3.1; Tables 1-2)
		(*c*) If relevant, consider translating estimates of relative risk into absolute risk for a meaningful time period	N/A	N/A

Continued on next page

Other analyses	17	Report other analyses done—eg analyses of subgroups and interactions, and sensitivity analyses	5,6,11	Results (sections 3.1-3.3)
**Discussion**				
Key results	18	Summarise key results with reference to study objectives	12-13	Discussion (paragraph 1)
Limitations	19	Discuss limitations of the study, taking into account sources of potential bias or imprecision. Discuss both direction and magnitude of any potential bias	15	Discussion (paragraph 6)
Interpretation	20	Give a cautious overall interpretation of results considering objectives, limitations, multiplicity of analyses, results from similar studies, and other relevant evidence	12-15	Discussion (all text)
Generalisability	21	Discuss the generalisability (external validity) of the study results	15	Discussion (paragraph 6)
**Other information**				
Funding	22	Give the source of funding and the role of the funders for the present study and, if applicable, for the original study on which the present article is based	16-17	Funding (all text)

*Give information separately for cases and controls in case-control studies and, if applicable, for exposed and unexposed groups in cohort and cross-sectional studies.

Note: An Explanation and Elaboration article discusses each checklist item and gives methodological background and published examples of transparent reporting. The STROBE checklist is best used in conjunction with this article (freely available on the Web sites of PLoS Medicine at http://www.plosmedicine.org/, Annals of Internal Medicine at http://www.annals.org/, and Epidemiology at http://www.epidem.com/). Information on the STROBE Initiative is available at www.strobe-statement.org.
